# Long-Term Pain Characteristics and Management Following Minimally Invasive Spinal Decompression and Open Laminectomy and Fusion for Spinal Stenosis

**DOI:** 10.3390/medicina57101125

**Published:** 2021-10-18

**Authors:** Gilad J. Regev, Gil Leor, Ran Ankori, Uri Hochberg, Dror Ofir, Morsi Khashan, Ron Kedem, Zvi Lidar, Khalil Salame

**Affiliations:** 1 The Department of Neurosurgery, Tel-Aviv Sourasky Medical Center, Tel Aviv 6423906, Israel; ranan@tlvmc.gov.il (R.A.); drorofir.2y@gmail.com (D.O.); morsi.kh@gmail.com (M.K.); lidar.zvi@gmail.com (Z.L.); khalils@tlvmc.gov.il (K.S.); 2 Sackler Faculty of Medicine, Tel Aviv University, Tel-Aviv 6997801, Israel; gilleor23@gmail.com; 3 Academic Branch, Medical Corps, Israel Defense Force, Tel Aviv 02149, Israel; ron.kedem56@gmail.com; 4 The Department of Pain Medicine, Division of Anesthesiology, Tel Aviv Sourasky Medical Center, Tel Aviv 6423906, Israel; urih@tlvmc.gov.il

**Keywords:** minimally invasive, spinal decompression, laminectomy, fusion, clinical outcomes

## Abstract

*Background and Objectives*: To compare the long-term pain characteristics and its chronic management following minimally invasive spinal (MIS) decompression and open laminectomy with fusion for lumbar stenosis. *Materials and Methods*: The study cohort included patients with a minimum 5-year postoperative follow-up after undergoing either MIS decompression or laminectomy with fusion for spinal claudication. The primary outcome of interest was chronic back and leg pain intensity. Secondary outcome measures included pain frequency during the day, chronic use of non-opioid analgesics, narcotic medications, medical cannabinoids, and continuous interventional pain treatments. *Results*: A total of 95 patients with lumbar spinal stenosis underwent one- or two-level surgery for lumbar spinal stenosis between April 2009 and July 2013. Of these, 50 patients underwent MIS decompression and 45 patients underwent open laminectomy with instrumented fusion. In the fusion group, a higher percentage of patients experienced moderate-to-severe back pain with 48% compared to 21.8% of patients in the MIS decompression group (*p* < 0.01). In contrast, we found no significant difference in the reported leg pain in both groups. In the fusion group, 20% of the patients described their back and leg pain as persistent throughout the day compared to only 2.2% in the MIS decompression group (*p* < 0.05). A trend toward higher chronic dependence on analgesic medication and repetitive pain clinic treatments was found in the fusion group. *Conclusions*: MIS decompression for the treatment of degenerative spinal stenosis resulted in decreased long-term back pain and similar leg pain outcomes compared to open laminectomy and instrumented fusion surgery.

## 1. Introduction

The proportion of the elderly population in developed countries has been rising in recent years and is expected to continue to increase in the future. As a result, age-related degenerative diseases have become a common cause for those seeking medical treatment [1]. Among these conditions, degenerative lumbar spinal stenosis is the most common indication for spinal surgery in the older population [2]. Recent studies showed an increase in the number of spinal surgery procedures over the past few decades [3]. Furthermore, there has been a shift toward more complex and expensive procedures that mostly include instrumentation and fusion [4,5]. Today, surgical options are largely divided into decompression only or decompression and fusion with instrumentation. Several studies now support the idea that in the absence of spinal instability, decompression alone is sufficient for the treatment of spinal stenosis, with the fusion being largely unnecessary [6,7]. However, there are those who still argue that the addition of an instrumented fusion in the setting of advanced degenerative changes of the discs and facet joints may result in some long-term benefits, even in the absence of a clear indication for fusion [8].

In recent years, minimally invasive spinal (MIS) decompression has gained popularity as an alternative surgical procedure for the treatment of lumbar spinal stenosis [9]. This approach significantly reduces soft tissue injury and intraoperative bleeding. Furthermore, this approach minimizes muscle striping of the vertebral posterior elements and preserves the posterior ligamentous structures [10,11]. Previous studies on outcomes following MIS spinal decompression found that patients experienced less immediate postoperative pain, were usually mobilized and discharged earlier, and had a significantly lower risk of infections and systemic complications compared to patients who underwent open spinal procedures [12,13,14].

Although previous studies found that long-term functional outcomes of MIS decompression are similar to open laminectomy [13], it remains unclear whether long-term postoperative outcomes of MIS decompression regarding back and leg pain and management are also comparable to those of open laminectomy with instrumented fusion. To the best of our knowledge, previous studies focused on standardized functional outcomes but did not focus specifically on chronic pain characteristics or its treatments.

The aim of the present study was to compare the long-term pain intensity, localization, frequency, and management of patients following MIS decompression and open laminectomy with fusion for the treatment of lumbar stenosis.

## 2. Methods

This study was approved by our local Institutional Review Board and all patients provided informed consent before conducting the follow-up by phone interview. We retrospectively collected medical records on patients that underwent one- or two-level lumbar spine surgery between April 2009 and July 2013. All the patients that were referred to our unit completed a trial of various conservative treatments for at least six months. Treatments included oral analgesics, spinal injection, and radiofrequency combined with physiotherapy.

At our institute, the Spine Service works in close collaboration with both the Pain Management and the Physical Medicine and Rehabilitation Departments. A multi-disciplinary team routinely evaluates patients prior to referral for surgery.

Inclusion criteria included low back pain and radicular pain; neurogenic claudication, as defined by back and leg pain limiting ambulation and/or standing tolerance; and radiological evidence of degenerative lumbar stenosis with or without stable degenerative spondylolisthesis. Exclusion criteria included previous spine surgeries; three or more operated levels; the presence of a fracture or oncological pathology in the spine; and the presence of unstable spondylolisthesis, as shown in flexion–extension lumbar X-rays. The MIS group consisted of patients who underwent minimally invasive bilateral laminectomy via a unilateral approach, and the fusion group consisted of patients that underwent conservative open laminectomy with instrumentation and fusion. Patients’ pre-operative symptoms and spinal pathology, as evaluated using pre-operative MRI studies, were similar in both groups and the surgical procedure was elected solely on the basis of the treating surgeon’s preference and expertise. Patients were referred in an arbitrary manner to either a surgeon who is experienced in MIS or to a surgeon who is accustomed to performing laminectomies with an instrumented fusion to all degenerative cases who presented with significant degenerative changes in the discs and facet joints, even without radiological evidence of segmental instability. The rate of additional spinal surgeries following the initial surgery was also assessed between the groups.

Out of 401 eligible patients from our medical records, 194 met the inclusion criteria. Phone interviews were done at least 5 years following surgery. Of these, 95 patients (50 patients in the MIS group and 45 in the fusion group) were available for follow-up and were included in the analysis. The remaining 99 patients were unavailable or refused to collaborate with the researchers when approached. The follow-up questionnaire assessed their chronic back and leg pain intensity and management. Patients were asked to assess their average pain intensity over the last year. For simplicity, we opted to use the Verbal Descriptor Scale (VDS) pain assessment over the standard ten-grade Numerical Rating Scale (NRS). The VDS includes 4 response options. Pain intensity was graded as follows: grade 1—no pain (NRS 0), grade 2—mild pain (NRS 1–3), grade 3—moderate pain (NRS 4–6), and grade 4—severe pain (NRS 7–10) [15].

Pain frequency was assessed and categorized as follows: 1—experienced pain less than once a week, 2—experienced pain at least once a week, 3—experienced pain every day for less than one hour, 4—experienced pain for more than an hour a day, and 5—experienced constant pain throughout the day [16,17]. Lastly, patients were asked whether they were currently dependent on chronic pain treatments, including non-opioid analgesics, narcotic medications, medical cannabinoids, and/or continuous interventional pain treatments, by a pain clinician.

The telephone interviews were conducted by two of the authors (R.A. and G.L.).

### 2.1. Surgical Technique

All procedures were performed in a single medical center by senior spinal surgeons who were well experienced in MIS surgeries. MIS decompression procedures were done routinely under general anesthesia using an 18 or 20 mm tubular retractor system (METRx; Medtronic Sofamor Danek, Memphis, TN, USA) and a surgical microscope. Surgery was performed using a unilateral approach, with either an ipsilateral or bilateral canal decompression while preserving the spinal midline structures, including the spinous process, supraspinous, and intra-spinous ligaments.

### 2.2. Statistical Analysis

The data were analyzed using the IBM Corporation and other (8) 1989, 2019, Statistical Package for Social Sciences (SPSS, Inc., Chicago, IL, USA) version 24.0. The chi-squared and Fisher’s exact tests were used to evaluate the categorical variables. The non-parametric Mann–Whitney test was used to compare the continuous variables between the groups. A *p*-value less than 0.05 was considered statistically significant.

## 3. Results

The mean age was 69.9 ± 11.7 years in the MIS group and 63 ± 13 years in the fusion group (*p* > 0.01). The male/female ratio was similar in both groups (*p* = 0.2). The median follow-up period was 6.2 ± 1.6 years. No statistically significant differences were found in the number of operated levels, specific level of surgery and prevalence of degenerative spondylolisthesis, the clinical or radiological characteristics of patients, or the rate of lumbar spine reoperation in both groups (Table 1).

### 3.1. Back and Leg Pain Intensity and Frequency

The fusion group was found to have a higher percentage of patients that experienced intermediate-to-severe back pain, with 48% compared to 21.8% of patients in the MIS decompression group (*p* < 0.01) (Table 2).

**Figure 1:**
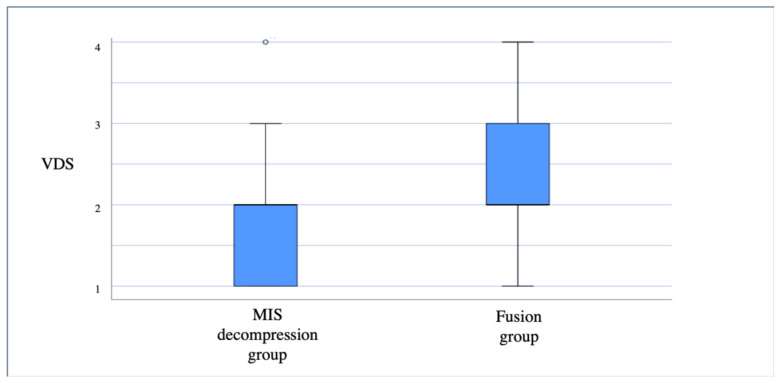
Distribution of the reported Verbal Descriptor Scales (VDS) for back pain for the MIS decompression and fusion groups.

In the MIS decompression group, 41.3% of the patients reported no back pain and 37% reported mild back pain compared to only 18% and 34%, respectively, in the fusion group (*p* < 0.01) (Figure 1).

**Figure 2:**
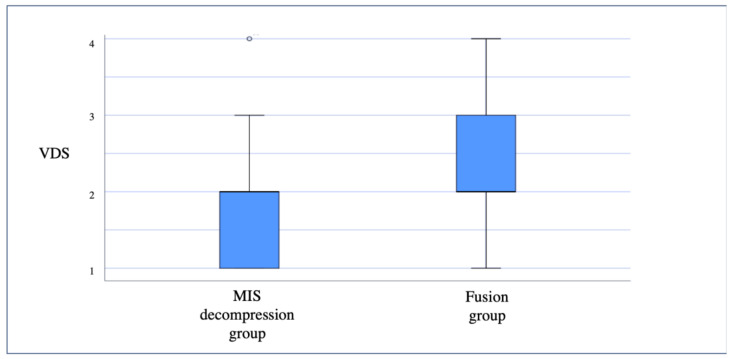
Distribution of the reported Verbal Descriptor Scales (VDS) for leg pain for the MIS decompression and fusion groups.

In contrast with back pain, we found no significant difference in the reported leg pain in both groups. In the MIS decompression group, 43.5% of patients reported intermediate-to-severe leg pain compared to 52% in the fusion group. However, 28.3% of the patients in the MIS decompression group did not experience any radicular pain compared to only 24% in the fusion group, though this difference did not reach statistical significance (*p* = 0.2) (Figure 2).

**Figure 3:**
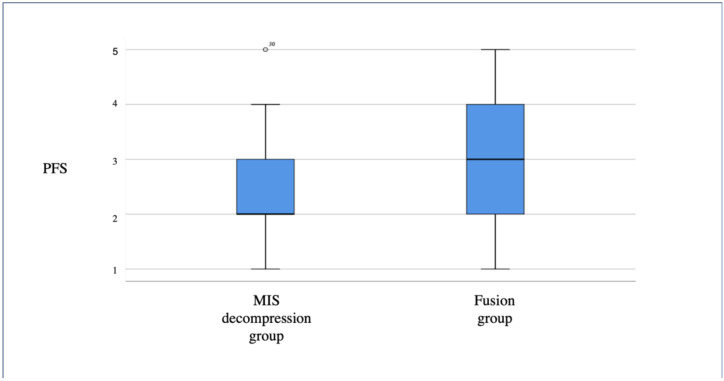
Distribution of back and leg pain frequency for the MIS and fusion groups.

**Figure 4:**
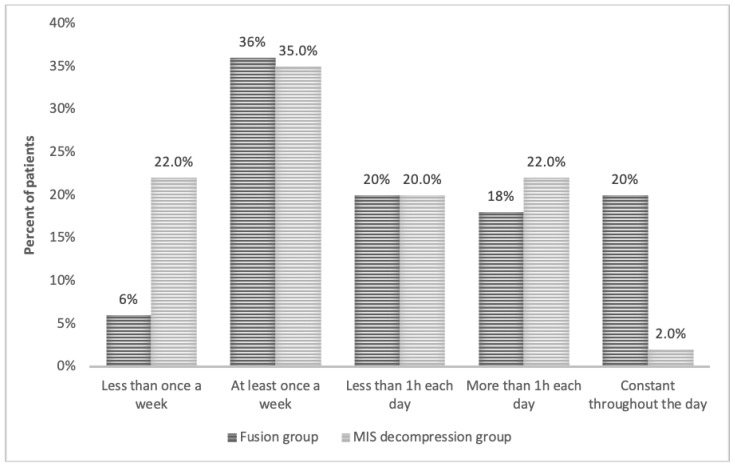
Pain frequency among the MIS and fusion groups.

In the fusion group, 20% of the patients described their back and leg pain as persistent throughout the day compared with only 2.2% in the MIS decompression group (*p* < 0.05) (Figure 2). Furthermore, in the MIS group, 21.7% of the patients in the decompression group reported having a low pain frequency of “less than once a week” compared with only 6% in the fusion group (*p* < 0.05) (Figure 3 and Figure 4).

### 3.2. Use of Pain Medication

**Figure 5:**
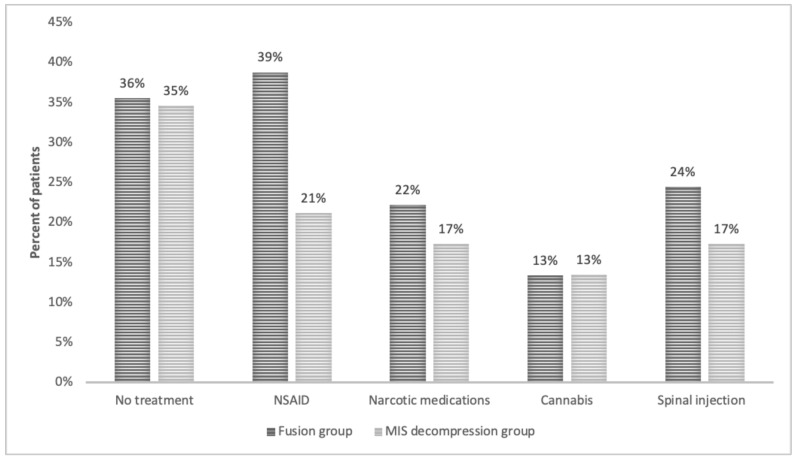
Chronic pain treatments among the MIS and fusion groups.

A trend toward a higher dependence on analgesic medications and continuous pain clinic treatments was observed in patients of the fusion group, but this trend was statistically insignificant. In both groups, one-third of the patients reported not using any analgesic medications on a regular basis (35.6% in the fusion group vs. 34.6% in the MIS group) (*p* = 0.6). In the fusion group, 22.2% of patients reported chronic use of some narcotic medications and 38.8% reported chronic use of non-opioid analgesics. In comparison, only 17.3% of patients in the MIS group reported chronic use of narcotic medications (*p* = 0.5) and 21.2% reported chronic use of non-opioid analgesics (*p* = 0.07). Moreover, 24.4% of patients in the fusion group reported that they continued treatment in pain clinics compared to 17.3% in the MIS group (*p* = 0.4). Thirteen percent of the patients in both groups reported using cannabis-based medication for their pain (*p* = 1) (Figure 5).

## 4. Discussion

Spinal stenosis, with its insidious onset and chronicity, is a common cause of disability among the elderly population [18]. Conservative measures are recommended as a first step and are mostly successful when applied correctly [19]. Lumbar spine surgery, in its various forms, is typically recommended for those patients who are deemed refractory to conservative measures or when impending neurological damage is recognized.

This study compared two groups of patients that failed conservative treatment and underwent either MIS lamino-foraminotomy or open laminectomy and fusion for the treatment of degenerative lumbar stenosis. Although the underlying pathology and presenting symptoms of patients in both groups were similar, the surgical approaches that were used for their treatments were based on profoundly different strategies. In the fusion group, the surgeon opted to perform a wide laminectomy and bilateral facetectomy, necessitating the addition of instrumentation and fusion to address the risk of iatrogenic instability. On the other hand, in the MIS decompression group, the surgical procedure was performed with attention toward minimizing the damage to the paraspinal soft tissue and bony structures in an attempt not to change the native biomechanical characteristics of the spinal column, even when pathologies, such as degenerative spondylolisthesis and scoliosis, were noted.

Previous studies found that following lumbar laminectomy, improvement in neurogenic claudication and radicular pain tended to decrease with time. This phenomenon was attributed to either same level re-stenosis or adjacent level stenosis [18,20,21]. Performing an open and wide laminectomy has the potential benefit of achieving robust decompression that can potentially reduce the risk of spinal re-stenosis and recurrent radicular pain. As such, our expectation was that long-term radicular pain scores would be superior in the fusion group compared with the MIS group. However, our results did not reveal any statistical difference with regard to leg pain between the groups. Therefore, it seems that performing a unilateral approach decompression that focused on limited medial facetectomy resulted in comparable clinical outcomes, even during a long-term follow-up [22]. This finding was also supported by previous studies that reported good favorable clinical mid-term and long-term outcomes following MIS bilateral decompression via a unilateral approach [12,21,23]. Musluman et al. found that MIS spinal decompression using a unilateral approach resulted in a significant improvement in patients’ functional outcomes. Moreover, those patients who were diagnosed with degenerative spondylolisthesis did not have significant changes in their spinal alignment in a two-year follow-up [13]. Kelleher et al. evaluated patients’ outcomes using a cohort of consecutive cases that were operated by a single surgeon over 5 years. They concluded that for leg-dominant symptoms, MIS lumbar decompression alone was a clinically effective procedure in the majority of patients, including those with degenerative spondylolisthesis or scoliosis [23].

The addition of an instrumented fusion should have a similar potential in improving patients’ long-term back pain. The immobilization of the arthritic facet joints and degenerative discs that often accompany spinal stenosis should reduce mechanical back pain. Earlier studies that followed up patients undergoing open laminectomy found that some of the patients experienced worsening of their axial back pain, which was attributed to iatrogenic spinal instability following the bilateral violation of the facet joints [6,7]. However, recent studies indicated that the addition of fusion to decompression may not provide any advantage for the treatment of lumbar stenosis. Forsth et al. conducted a prospective randomized study that compared patients that were treated with either decompression plus fusion or open decompression alone for lumbar spinal stenosis. They found that the addition of fusion did not result in better clinical outcomes after 2 and 5 years [6]. Similarly, Thomas et al. analyzed the Canadian Spine Outcomes and Research Network (CSORN) prospective database for consecutive spine surgery cases of degenerative lumbar stenosis. They reported that the addition of fusion to decompression provided no advantage compared to decompression alone for the treatment of patients with neurogenic claudication secondary to lumbar stenosis without spondylolisthesis or deformity [7]. However, to the best of our knowledge, such a comparison between MIS decompression and open decompression with fusion has not been published to date.

Our results indicated that, in comparison with open decompression with fusion, MIS decompression may have superior long-term outcomes in terms of back pain intensity and frequency. In the fusion group, a significantly greater proportion of patients reported experiencing long-term intermediate-to-severe back pain compared with patients in the MIS decompression group. This finding was consistent with the higher percentage of patients in the MIS decompression group who reported having no back pain and a low frequency of pain. Although previous studies failed to show any differences in post-operative back pain when comparing open surgery to MIS decompression [11,14,20,24,25], it is possible that in our study, minimizing damage to the paraspinal muscles and preservation of the ligamentous midline helped patients retain better long-term back muscle function, which resulted in less back pain.

The use of narcotic medication following surgical interventions, including spine surgery, may predispose patients to chronic opioid use and abuse. Several studies focused on identifying risk factors that predispose patients to this opioid dependency. Some studies found that performing instrumented spinal fusion was one of the leading risk factors that predicted prolonged opioid prescriptions in opioid-naive patients [26,27,28]. Moreover, Schoenfeld et al. showed that low-intensity surgical procedures were associated with a higher likelihood of discontinuing opioid use [29].

We also found a similar trend when analyzing the chronic use of pain medication in both groups. A higher percentage of patients from the fusion group was dependent on the chronic use of narcotic and non-opioid pain medications, as well as a higher percentage of patients that continued injection treatments at the pain clinic. However, this trend did not reach statistical significance, possibly due to the size of our cohort. Interestingly, we did not see any difference in the chronic use of cannabis-based medications between the groups.

There are a few limitations to our study. First, this is a retrospective analysis of only half of the patients that met the inclusion/exclusion criteria due to lack of follow-up. Furthermore, the average follow-up time and the mean age of the patients were significantly different between the groups. The patients in the MIS decompression group were significantly older than in the fusion group and the mean follow-up time was longer in the MIS decompression group. However, since the minimum follow-up time was 5 years from surgery, we can reasonably argue that the pain parameters obtained at the actual time of follow-up represented the chronic pain condition of the patients. In addition, we would expect that the older age of patients in the MIS decompression group would have a negative effect on their general physical condition and pain levels. As such, these differences between the groups actually supported our findings since the MIS decompression group was found to have better pain-related outcomes compared to the fusion group. It is still possible that a randomized controlled trial on a large-scale (national) cohort could yield different results. Lastly, we were unable to attain detailed and reliable pre-operative pain measurements of the patients and compare them to their current pain levels. However, the strength of our study relied on the analysis of multiple variables in order to evaluate not only the pain intensity but also its frequency and different treatments, thus allowing for a better understanding of the pain’s impact on the patients’ lives. Such variables are usually not available when using large-scale spine registries but may provide additional insight, both from therapeutic and cost-effectiveness perspectives, in order to improve the quality of spinal health care.

In conclusion, our results indicated that when comparing open laminectomy with fusion and MIS decompression surgeries for the treatment of lumbar spinal stenosis, we found a higher intensity of back pain and a trend toward increased chronic use of analgesic medication and interventional pain treatments in the fusion group. Additionally, performing open laminectomy and instrumented fusion provided no advantage compared to minimally invasive decompression for the treatment of patients’ radicular pain with stable lumbar canal stenosis. Future prospective studies are necessary to validate the specific advantages of minimally invasive techniques for spine surgery.

## Figures and Tables

**Table 1:** Demographic data and radiological and surgical characteristics of both groups.

	MIS Decompression Group	Fusion Group	*p*-Value
Age (years) *	69.88 ± 11.7	63.6 ± 13	0.0092
**Male/female**	22/28	15/30	0.4
**Follow-up (years)**	7.82 ± 0.25	5.89 ± 0.09	>0.001
**L2-3**	3 (6%)	0	0.05
**L3-4**	15 (30%)	10 (21.7%)	0.5
**L4-5**	34 (68%)	30 (65.2%)	0.8
**L5-S1**	3 (6%)	18 (39.1%)	0.2
**Degenerative spondylolisthesis**	9 (18%)	8 (17.8%)	0.8
**One-level surgery**	47 (90.4%)	33 (71.7%)	0.7
**Two-level surgery**	5 (9.6%)	13 (28.3%)	0.3
**Reoperation and other lumbar spine surgeries**	11 (22%)	13 (29%)	0.65

* Values are expressed as mean ± SD.

**Table 2:** Back and leg pain intensity and frequency in both groups.

		Surgery Type		*p*-Value
		MIS Decompression Group (*n* = 46)	Fusion Group (*n* = 50)	
Back pain	None	19 (41.3%)	9 (18%)	0.0029
	Mild	17 (37%)	17 (34%)	
	Moderate	9 (19.6%)	12 (24%)	
	Severe	1 (2.2%)	12 (24%)	
Leg pain	None	13 (28.3%)	12 (24%)	0.3116
	Mild	13 (28.3%)	12 (24%)	
	Moderate	15 (32.6%)	13 (26%)	
	Severe	5 (10.9%)	13 (26%)	
Pain frequency	Less than once a week	10 (21.7%)	3 (6%)	0.0203
	At least once a week	16 (34.8%)	18 (36%)	
	Less than 1 h each day	9 (19.6%)	10 (20%)	
	More than 1 h each day	10 (21.7%)	9 (18%)	
	Constant	1 (2.2%)	10 (20%)	

## Data Availability

Not applicable.
